# Use of speckle-tracking echocardiography–derived strain and systolic strain rate measurements to predict rejection in transplant hearts with preserved ejection fraction

**DOI:** 10.1186/s12872-018-0980-4

**Published:** 2018-12-22

**Authors:** Andrew S. Tseng, Umama S. Gorsi, Sergio Barros-Gomes, Fletcher A. Miller, Patricia A. Pellikka, Alfredo L. Clavell, Hector R. Villarraga

**Affiliations:** 1 Department of Internal Medicine, Mayo Clinic, 13400 E Shea Blvd, Scottsdale, AZ 85259 USA; 2 Department of Cardiovascular Diseases, Mayo Clinic, Rochester, Minnesota, 200 First St SW, Rochester, MN 55905 USA

**Keywords:** Echocardiography, Imaging, Rejection, Heart transplant, Strain

## Abstract

**Background:**

Noninvasive diagnosis of allograft rejection in heart transplant recipients is challenging. The utility of 2-dimensional speckle-tracking echocardiography (2D-STE) to predict severe rejection in heart transplant recipients with preserved left ventricular ejection fraction (LVEF) was evaluated.

**Methods:**

Adult heart transplant patients with preserved LVEF (> 55%) and severe rejection by biopsy (Rejection Grade ≥ 2R) or no rejection between 1997 and 2011 at the Mayo Clinic in Rochester, Minnesota were evaluated. Transthoracic echocardiography was performed within 1 month of the biopsy. LV global longitudinal and circumferential strain and strain rates (GLS, GLSR, GCS, and GCSR) were analyzed retrospectively.

**Results:**

Of 65 patients included, 25 had severe rejection and 40 were normal transplant controls without rejection. Both groups had more men than women (64 and 75%, respectively). Baseline clinical variables were similar between the groups. Both groups had normal LVEF (64.3% vs 64.5%; *P* = .87). All non-strain echocardiographic variables were similar between the 2 groups. Strain analysis showed significantly increased early diastolic longitudinal strain rate (*P* = .02) and decreased GCS (*P* < .001) and GCSR (*P* = .02) for the rejection group compared with the control group. The area under the receiver operating characteristic curve for GCS was 0.77. With a GCS cutoff of − 17.60%, the sensitivity and specificity of GCS to detect severe acute rejection were 81.8 and 68.4%, respectively.

**Conclusions:**

2D-STE may be useful in detecting severe transplant rejection in heart transplant patients with normal LVEF.

## Background

Heart transplantation is the treatment of choice for many patients with end-stage heart failure. Improvement in long-term survival has been recognized in the past several decades as a consequence of careful recipient and donor selection, advances in immunosuppression, and management of opportunistic infections. A 2015 report by the Organ Procurement and Transplantation Network and the Scientific Registry of Transplant Recipients documented the overall survival rate after heart transplant in the United States as 82% after 1 year and approximately 69% after 5 years [1]. As such, detecting severe acute heart transplant rejection is imperative for increasing the survival of heart transplant patients. Currently, the diagnostic standard for organ rejection is myocardial biopsy, an invasive procedure that has numerous potential complications including carotid puncture, prolonged bleeding, arrhythmias, and myocardial perforation [2]. The risk of major complications from this invasive procedure is estimated to be between 0.09 and 5.2% [3].

Echocardiography is an important noninvasive tool to evaluate graft size and function, given its portability and availability. The use of 2-dimensional speckle-tracking echocardiography (2D-STE), in particular, to measure strain and systolic strain rate is important for evaluating myocardial mechanical dysfunction [4, 5]. 2D-STE determines strain and systolic strain rate from myocardial motion derived from the echocardiogram. In otherwise normal heart transplant patients, systolic strain indices obtained from 2D-STE were determined to be sensitive markers of myocardial dysfunction [6]. Left ventricular (LV) mechanical function in heart transplant patients and non-transplant patients has previously been analyzed using 2D-STE, which showed decreased strain in transplant patients. Saleh et al. [7] previously reported normal LV mechanical function and synchrony values by 2D-STE in transplanted hearts with preserved ejection fraction (EF). Previous studies using 2D-STE have shown that various indices of LV radial, circumferential, longitudinal and torsional strain may be useful in detecting heart transplant rejection [8–12].

The aim of this study was to evaluate the use of 2D-STE–derived strain measurements as a noninvasive clinical prediction tool in a specific subset of patients with severe acute rejection in heart transplant patients with preserved LVEF.

## Methods

### Study population

The study population consisted of adult patients (age greater than or equal to 18 years) who underwent heart transplantation at Mayo Clinic, Rochester, Minnesota, from January 1, 1997, through December 31, 2011. Patients were retrospectively identified by a search of our institutional patient database. All heart transplant patients during this time period who experienced severe biopsy-confirmed cell-mediated rejection (2R or greater) based on the 2004 International Society for Heart and Lung Transplantation (ISHLT) grading system [13] and who had LVEF greater than 55% were included in this study. Patients with known transplant vasculopathy were excluded. Transthoracic echocardiography was performed within 1 month prior to the biopsy. Control participants included heart transplant patients without transplant vasculopathy by angiography or intravascular ultrasonography, with no evidence of rejection by routine surveillance endomyocardial biopsy and normal echocardiography findings at one-year post-transplantation follow-up. Patients with abnormal renal function, significant valvular disease, or uncontrolled hypertension were excluded from the study. The electronic medical records were reviewed for demographic characteristics, along with clinical and echocardiographic data. Pertinent laboratory values, medications, and other clinical history were obtained at the time of echocardiography. All participants provided written informed consent. The study protocol was approved by the Mayo Clinic Institutional Review Board.

### Standard echocardiography

Comprehensive 2D echocardiography was performed using a Vivid E9 Ultrasound System (GE Healthcare) with a 2.5- to 4.0-MHz transducer, as part of the recommendations from the American Society of Echocardiography [14]. The modified Quinones equation or volumetric biplane Simpson method, or both, as appropriate, was used to quantify LVEF. Offline 2D-STE analysis (Velocity Vector Imaging) was performed retrospectively. Clinical, survival, and echocardiographic data were masked to the reviewers.

### Speckle-tracking echocardiography

Three consecutive heart cycles from each of the apical views (apical 4-chamber, 3-chamber, and 2-chamber) were obtained for longitudinal strain analysis, including global longitudinal strain (GLS), global longitudinal strain rate (GLSR), and early diastolic global longitudinal strain rate (GLSRe). Short-axis views at the midpapillary level were obtained for circumferential strain analysis, including global circumferential strain (GCS) and global circumferential strain rate (GCSR).

Strain measurements were performed offline and analyzed with syngo Velocity Vector Imaging software (Siemens Medical Solutions USA, Inc) after transthoracic echocardiography evaluation. The LV wall was divided into 17 segments, and the tracing was performed using a 1-beat cycle starting at mid or end systole. The region of interest was adjusted to cover at least 90% of the myocardial wall thickness. Visual analysis was done first for endomyocardial tracking and, if required, manually adjusted. The epicardial border was then traced to include the entire myocardial thickness and was readjusted as needed. No segments were excluded for 2D-STE analyses. Clinical, survival, and echocardiographic data were masked to the reviewers.

### Statistical analysis

Continuous variables are presented as mean (SD); categorical variables are presented as number (percentage of total). Continuous data were analyzed with a *t* test or a Wilcoxon rank sum test when assumptions were not met. Paired *t* tests were used to compare means for GLS and segmental longitudinal strain between groups. Categorical variables were analyzed using either the Pearson χ^2^ or Fisher exact test. Receiver operating characteristic analysis was used to evaluate accuracy of strain and systolic strain rates to detect rejection.

For interobserver variability, two independent investigators (A.S.T. and S.B.-G.) to whom the initial results were masked assessed strain in 6 randomly selected patients. For intraobserver variability, repeat assessment of 2D-STE examination in 6 randomly selected patients by the same investigator was performed. To assess agreement, the concordance correlation coefficient (CCC) and Bland-Altman analysis, and calculation of bias (mean [SD] difference between 2 measurements) and of differences in individual pairs of measurements (limits of agreement) were used. Spearman rank correlation coefficient was also calculated.

Statistical analysis was performed using 2 commercially available software packages: JMP 12.0 (SAS Institute Inc) and statistical software version 12 (MedCalc Software 5). All probability values were 2-sided, and a *P*-value <.05 was considered statistically significant.

## Results

### Study population

A total of 65 heart transplant patients were included, 25 with severe rejection and 40 controls without rejection. Baseline demographics, risk factors, and clinical variables are summarized in Table 1. The mean (SD) age was 48.5 (13.5) years in the rejection group and 53.2 (11.5) years in the control group. There were more men than women in both groups (64 and 75%, respectively). All baseline variables, including body mass index, body surface area, hypertension, low-density lipoprotein cholesterol, diabetes mellitus, hypertension, heart rate, and hyperlipidemia, were similar in both groups. There were no significant differences in medication usage, including immunosuppression, prior prednisone use in the preceding 12 months, angiotensin-converting enzyme inhibitors, beta-blockers and statins. Eighty percent of patients had non-ischemic dilated cardiomyopathy as their heart transplant indication. In regards to symptoms, of the patients with severe rejection, 19 patients (76%) were asymptomatic at the time of diagnosis of severe rejection. The most frequently reported symptoms included lower extremity edema, dyspnea, dizziness, fatigue and headaches.

**Table 1:** Patient characteristics

Characteristic	Group^a^		*P*
	Control (*n* = 40)	Rejection (*n* = 25)	
Age, y	53.2 (11.5)	48.4 (13.5)	.15
Time since transplant, y	0.98 (0.08)	1.13 (1.3)	.47
Men	30 (75)	16 (64)	.34
BMI, kg/m^2^	25.96 (5.0)	25.96 (5.9)	.99
BSA, m^2^	1.94 (0.26)	1.87 (0.25)	.26
Hypertension	17 (44)	12 (45)	.73
LDL cholesterol, mg/dL	103.0 (32.47)	104.9 (39.15)	.57
Diabetes mellitus	8 (20)	3 (12)	.40
SBP, mm Hg	126.3 (15.3)	126.5 (15.5)	.95
DBP, mm Hg	76.3 (11.3)	79.1 (10.0)	.30
Heart rate, beats/min	90.2 (11.2)	94.5 (11.4)	.15
Hyperlipidemia	20 (50)	16 (64)	.27
QRS duration, ms	106.6 (19.5)	103.7 (25.6)	.61
Cyclosporine, n (%)	16 (40)	14 (56)	.21
Tacrolimus, n (%)	7 (18)	5 (20)	.84
Sirolimus, n (%)	19 (48)	9 (36)	.34
Azathioprine, n (%)	8 (20)	4 (16)	.69
Mycophenolate mofetil, n (%)	25 (63)	21 (84)	.07
Prednisone in last 12 months, n (%)	38 (95)	21 (84)	.14
ACE-I/ARB, n (%)	16 (40)	6 (24)	.19
Beta-blocker, n (%)	5 (13)	2 (8)	.54
Statin, n (%)	20 (50)	16 (64)	.27

*Abbreviations: BMI* body mass index, *BSA* body surface area, *DBP* diastolic blood pressure, *LDL* low-density lipoprotein, *SBP* systolic blood pressure

^a^Values are mean (SD) or No. of patients (%)

### Baseline echocardiographic and hemodynamic evaluation

Both groups had preserved and similar mean (SD) LVEF (64.3% [5.2%] rejection group vs 64.5% [3.7%] control; *P* = .87). LV end-diastolic diameter (45.9 [4.6] mm vs 46.3 [3.7] mm; *P* = .75), ratio of early and late diastolic waves of mitral inflow, or *E/A ratio* (2.21 [1.03] vs 1.97 [0.70]; *P* = .51), and deceleration time of mitral inflow, or *DT* (180.3 [25.5] ms vs 169.0 [27.7] ms; *P* = .23) were not significantly different between the rejection and control group, respectively. Table 2 summarizes the echocardiographic data for both groups.

**Table 2:** Conventional and Strain Echocardiographic Variables

Variable	Group^a^		*P*
	Control (*n* = 40)	Rejection (*n* = 25)	
LVEF, %	64.5 (3.7)	64.3 (5.2)	.87
LV EDD, mm	46.3 (4.2)	45.9 (4.6)	.75
LV ESD, mm	29.1 (3.2)	28.7 (2.8)	.59
LV IVSd, mm	11.3 (1.8)	10.9 (1.3)	.33
LV PWd, mm	11.0 (1.5)	11.0 (2.1)	.96
Mean LVWT, mm	11.1 (1.5)	10.9 (1.5)	.65
LVMI 2D, g/m^2^	95.3 (21.1)	98.0 (22.4)	.63
E/A ratio	1.97 (0.70)	2.21 (1.03)	.51
DT, ms	169.0 (27.7)	180.3 (25.5)	.23
e′_sep_, cm/s	0.086 (0.023)	0.093 (0.022)	.26
E/e′_sep_ ratio	11.0 (4.3)	11.2 (2.7)	.89
GCS, %	−19.90 (4.25)	−15.74 (3.40)	< 0.001
GCSR, s^− 1^	− 1.28 (0.30)	− 1.09 (0.30)	.02
GLS, %	−13.63 (2.35)	−13.18 (3.32)	.52
GLSR, s^−1^	−0.82 (0.15)	−0.86 (0.24)	.46

*Abbreviations: DT* deceleration time of mitral inflow, *E/A ratio* ratio of early and late diastolic waves of mitral inflow, *E/e′*_*sep*_*ratio* ratio of early wave of mitral inflow and early diastolic tissue Doppler velocity at septal mitral annulus, *EDD* end-diastolic diameter, *ESD* end-systolic diameter, *e′*_*sep*_ early diastolic tissue Doppler velocity at septal mitral annulus, *IVSd* interventricular septal thickness, *LV* left ventricular, *LVEF* LV ejection fraction, *LVMI 2D* left ventricular mass index, *LVWT* LV wall thickness, *PWd* posterior wall thickness

^a^Values are mean (SD)

### Strain analysis

**Fig. 1:**
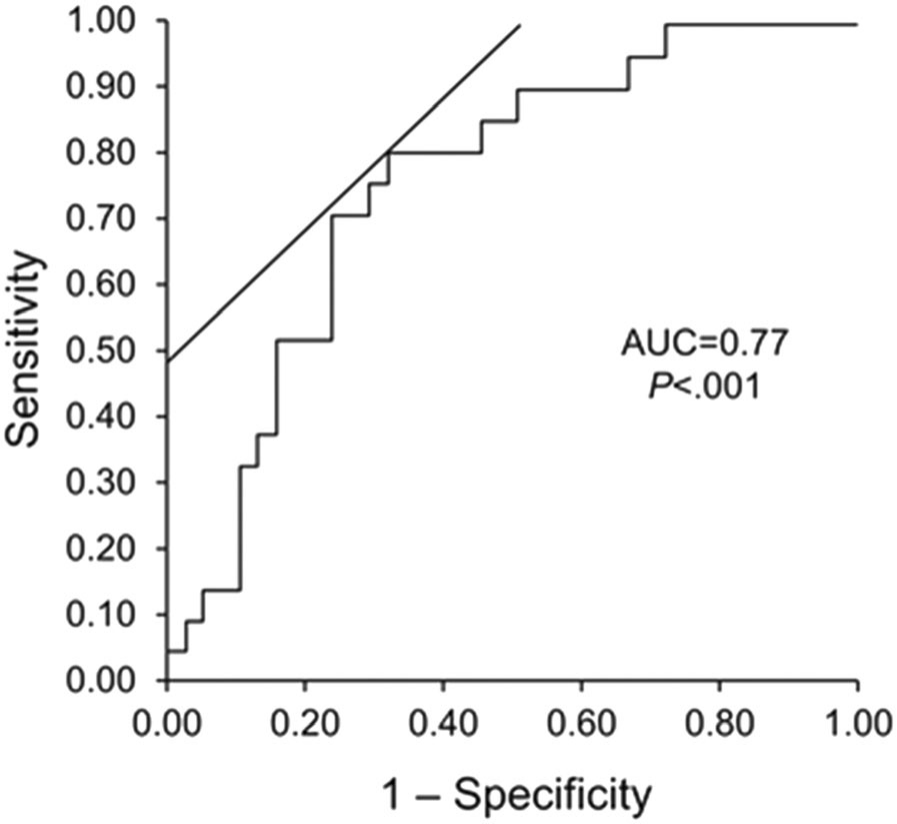
Global Circumferential Strain Area Under the Receiver Operating Characteristic Curve. With values less negative than a cutoff of − 17.60%, the sensitivity and specificity of GCS to detect severe acute rejection were 81.8 and 68.4%, respectively. Area under the curve (AUC) of the receiver operating characteristic analysis for global circumferential strain is 0.77 (*P* < .001)

There were no significant differences in mean (SD) GLS (− 13.18% [3.32%] vs − 13.63% (2.35%); *P* = .52) and GLSR (− 0.86 [0.24] s^− 1^ vs − 0.82 [0.15] s^− 1^; *P* = .46) between the rejection and control groups. Strain analysis showed significant differences in mean (SD) GLSRe (1.04 [0.28] s^− 1^ vs 0.80 [0.21] s^− 1^; *P* = .02), GCS (− 15.74% [3.40%] vs − 19.90% [4.25%]; *P* < .001), and GCSR (− 1.09 [0.30] s^− 1^ vs − 1.28 [0.30] s^− 1^; *P* = .02) between the rejection and control groups, respectively. The area under the receiver operating characteristic curve for GCS was 0.77. With values less negative than a cutoff of − 17.60%, the sensitivity and specificity of GCS to detect severe acute rejection were 81.8 and 68.4%, respectively (Fig. 1).

### Interobserver variability analysis

Interobserver data between 2 independent researchers showed strong agreement for GLS and GCS. For GLS, the Bland-Altman plots showed statistically significant minimal bias (mean [SD], − 0.17 [3.6]; *P* < .05) with relatively narrower limits of agreement (− 7.3 to 6.9 [1.96SD]); CCC, 0.57 (95% CI, 0.19–0.80); Pearson ρ (precision), 0.57; and bias correction factor Cb (accuracy), 0.99. For GCS, the Bland-Altman plots showed statistically significant minimal bias (mean [SD], 0.14 [4.3]; *P* < .05) with relatively narrower limits of agreement (− 4.4 to 4.6 [1.96SD]); CCC, 0.66 (95% CI, − 0.11-0.93); Pearson ρ (precision), 0.69; and bias correction factor Cb (accuracy), 0.95. Intraobserver variability analysis for GCS and GLS also showed agreement: CCC of 0.85 (95% CI, 0.65–0.94) and 0.97 (95% CI, 0.81–0.99), respectively.

## Discussion

The use of 2D-STE strain indices is becoming an important diagnostic tool in detecting acute rejection in heart transplant patients. We specifically evaluated the use of 2D-STE in a subset of patients who experienced severe acute rejection but had preserved LVEF. This group had decreased (ie, less negative) GCS as compared with heart transplant recipients without rejection. A cutoff value of–17.60% for GCS has a sensitivity and specificity of 81.8 and 68.4%, respectively (area under the receiver operating characteristic curve, 0.77), for its detection. GLS and GLSR were similar in both groups.

Our findings are consistent with those of previous animal and human studies. Pieper et al. [14] showed a decrease in GCS in the evaluation of cardiac transplant rejection in a rat model. Their study also showed that radial strain, peak systolic radial strain rate, and peak early diastolic radial strain rates were lower in rats with transplant rejection than in those without rejection. Using 2D-STE, they were able to detect LV dysfunction when conventional echocardiographic measures were unable to demonstrate a difference in systolic function between the 2 groups. Elied et al. [15] established the natural history of LV mechanics in transplanted hearts by 2D-STE and showed that persistently low GLS was a negative prognostic indicator in heart transplant patients. They also showed that failure of GLS to improve by 3 months after transplant is associated with a greater incidence of death and cardiac events. They demonstrated that heart transplant patients have LV regional and global dysfunction within the first 2 years of transplantation, which develops independently of transplant rejection, and that these findings are clinically detectable. In addition, significantly decreased circumferential strain was observed at 2 years after acute allograft rejection, as compared with those without rejection, although the values were comparable at the initial time of rejection [15].

Previously, our group showed that, in normal heart transplant patients without rejection compared with healthy persons without transplant, circumferential deformation indices were normal, but longitudinal deformation indices, including GLS (− 13.43% [2.39%] vs − 17.28% [2.30%]; *P* < .001) and GLSR (− 0.83 [0.15] s^− 1^ vs − 0.96 [0.13] s^− 1^; *P* < .001), were decreased. From these results in normal transplant patients it appears that longitudinal strain impairment occurs early as a result of insults that lead to LV dysfunction, such as LV remodeling (eg, ischemia-reperfusion injury, allograft vasculopathy, and previous allograft rejection) [7]. Pichler et al. showed that deformation values remain stable if LVEF is preserved. Therefore, it is assumed that strain values in normal heart transplant patients are decreased soon after transplantation and stay constant as long as allograft rejection does not develop [16].

Knowing these dynamic changes that occur in normal heart transplant patients and in those with rejection, we postulate that longitudinal strain is the first strain variable to be decreased in cardiac dysfunction. We assume that preserved circumferential strain might be responsible for maintaining cardiac function in transplant patients and that during acute rejection this compensatory mechanism is impaired and leads to graft dysfunction despite preserved LVEF. Further investigation should be performed to elucidate the mechanism of longitudinal and circumferential strain mechanics during acute rejection.

Contrary to our findings, previous studies have showed that acute transplant rejection was associated with a decrease in GLS and not GCS [9, 10, 12] (citations). Few studies evaluated the role of GCS in acute severe rejection and those that did evaluate GCS generally had few patients in the severe rejection group (ISHLT grade 2R or greater). Furthermore, the time since transplantation during each rejection event was variable and study dependent. In our cohort, severe rejection events were identified approximately one year post-transplantation, on average. While other mean times since transplantations for acute severe rejection events in Clemmensen et al. and Mingo-Santos et al. were 3 years and 2.5 years, respectively [10, 12]. Therefore, it is possible that measurement of circumferential strain may provide incremental benefit in evaluating acute rejection to detect more severe rejection, possibly in newer transplant grafts. Further investigation is required to confirm the diagnostic utility of GCS in severe acute rejection.

With the advancements in medical technology, it is important to diagnose acute subclinical rejection when LVEF and other conventional 2D echocardiographic parameters are normal. We have shown that strain and strain rate indices derived from 2D-STE may assist in the evaluation of heart transplant patients, especially if acute rejection is suspected despite preserved LVEF.

## Limitations

Our study has several limitations. The study population consisted of a small sample from a single center. Larger studies are necessary to further evaluate the validity of the use of strain and strain rate derived from 2D-STE in the evaluation of heart transplant graft function during severe acute rejection. Furthermore, significant variability between different image processing software exists. Therefore, there are limitations in creating universal cutoff values for strain parameters when different studies use different programs to perform tracings and calculate strain.. The retrospective study design without serial echocardiographic examinations limits the conclusions we were able to draw, particularly is it pertains to the longitudinal changes in strain parameters over time. Although we excluded patients with known transplant vasculopathy, we were unable to definitively ascertain whether patients had concomitant vasculopathy during the acute episode of rejection. Also, our study did not evaluate radial strain because higher interobserver variability has been previously reported in the literature [17]. Lastly, the present study also excluded pediatric heart transplant patients in whom the same mechanophysiology may not apply.

## Conclusion

In our study, GCS, which is initially preserved in normal transplant patients, is significantly decreased in patients with preserved LVEF and severe acute rejection, defined as grade 2R or greater using the 2004 ISHLT rejection grading system. 2D-STE can be a useful clinical tool in monitoring heart transplant patients for acute rejection. With increasing evidence of its clinical utility, this noninvasive technique may be useful in detecting rejection in heart transplant patients.

## Abbreviations

2D-STE: 2-dimensional speckle-tracking echocardiography
CCC: Concordance correlation coefficient
EF: Ejection fraction
GCS: Global circumferential strain
GCSR: Global circumferential strain rate
GLS: Global longitudinal strain
GLSR: Global longitudinal strain rate
GLSRe: Early diastolic global longitudinal strain rate
ISHLT: International Society for Heart and Lung Transplantation
LV: Left ventricular
